# Influence of maternal and perinatal factors on macronutrient content of very preterm human milk during the first weeks after birth

**DOI:** 10.1038/s41372-022-01475-6

**Published:** 2022-08-05

**Authors:** Cristina Borràs-Novell, Ana Herranz Barbero, Carla Balcells Esponera, Miriam López-Abad, Victoria Aldecoa Bilbao, Montserrat Izquierdo Renau, Isabel Iglesias Platas

**Affiliations:** 1 Neonatology Department. BCNatal – Centre de Medicina Maternofetal i Neonatologia de Barcelona. Hospital Clínic, Universitat de Barcelona, Barcelona, Spain; 2 Neonatology Department. BCNatal – Centre de Medicina Maternofetal i Neonatologia de Barcelona. Hospital Sant Joan de Déu, Universitat de Barcelona, Barcelona, Spain; 3 Neonatal Intensive Care Unit, Norfolk and Norwich University Hospital, Norwich, UK

**Keywords:** Malnutrition, Outcomes research

## Abstract

**Objective:**

To identify changes in macronutrient content of very preterm human milk associated with perinatal factors.

**Study design:**

Milk macronutrients were measured on weeks 1, 2, 4 and 8 with mid-infrared transmission spectrometers.

**Result:**

We assessed 625 samples (from 117 mothers and 130 very preterm infants). Average concentrations were: protein 1.3 ± 0.3 g/dl, carbohydrates 7.3 ± 0.6 g/dl, fat 3.7 ± 1.0 g/dl and energy 296.0 ± 41.0 kJ/dl (70.7 kcal/dl). Gestational age negatively correlated with protein (*rho*: −0.307, *p* < 0.001) and energy (r: −0.193, *p* = 0.003). Advanced maternal age, gestational age and intrauterine growth restriction were independently associated with milk protein content over the first 4 weeks (adjusted R^2^: 0.113, *p* = 0.002).

**Conclusion:**

These findings may help neonatologists identify patients fed Mother´s Own Milk who are at increased risk of poor postnatal growth.

## Introduction

Postnatal growth restriction is one of the most prevalent problems in preterm infants [1]. Around 50% of growth in very premature infants (VPI) is estimated to depend on nutritional support [2]. Following an initial period of parenteral nutrition, enteral feeding is provided as milk, with Mother´s Own Milk (MOM) as the best option [3], due to its strong association with a reduction in the incidence of some prematurity-related diseases (such as necrotizing entercolitis), and with a more favourable neurodevelopmental outcome [4–6] both of which might be related to an array of bioactive factors [7]. The last published guidance for enteral nutrition in preterm infants by the European Society of Paediatric Gastroenterology, Hepatology and Nutrition (ESPGHAN) [8] recommends an intake of 110–135 kcal/kg/day with between 3.5 and 4.0 (for patients weighing 1000–1800 g) and 4.0–4.5 g/kg/day (if under 1000 g) of protein. Despite all other clear benefits, the nutritional content of human milk is insufficient to meet these high demands, and fortification is required to increase the concentration of protein, calcium and phosphorus [9].

Macronutrient content of human milk is highly variable and it changes in association with time [10], maternal age, diabetes or obesity, multiple pregnancy or parity, neonatal birth weight or gender [11–15]. Most available information on this topic applies to lactation after term delivery, but more recent studies focus in the relationship between preterm milk composition and maternal, pregnancy and neonatal factors [11, 12, 16–19]. Disparity in methodological aspects and results make it complicated to draw firm conclusions [20]. Studies span different gestational ages, analyze single or pooled samples and use a variety of techniques to determine macronutrient content.

Nutrient variability in milk may cause cumulative deficits over time in VPI despite fortification and may play a role in poor postnatal growth [21, 22]. Although targeted fortification could help, it requires considerable economic and human resources [23]. Information on the impact of maternal, gestational or neonatal characteristics on milk macronutrient composition could help identify mother-infant pairs at higher risk of extrauterine growth restriction and move towards a more individualized nutritional support [24].

In this context, we aimed to measure macronutrient content of very preterm milk and to identify changes associated with time and maternal, gestational and neonatal characteristics.

## Methods

We designed an observational prospective bicentric cohort study. The study followed the reporting guidelines for observational studies (Strengthening the Reporting of Observational Studies in Epidemiology - STROBE) [25]. The protocol was approved by the local research ethics committees from Fundació Sant Joan de Déu (PIC-147-17) and Hospital Clínic de Barcelona (HCB/2016/0959).

Lactating mothers and their VPI (delivery at or before 32^0^ weeks of gestation) admitted to the Neonatal Unit between January 2018 and January 2020 were consecutively approached for inclusion in the study when milk production exceeded enteral feeding requirements. Mothers of newborns with congenital malformations, known chromosomal, genetic or metabolic anomalies or low chances of short-term survival as per judgment of the attending clinician were excluded. Parents of eligible neonates provided written informed consent. The study was performed in accordance with the Declaration of Helsinki.

**Fig. 1:**
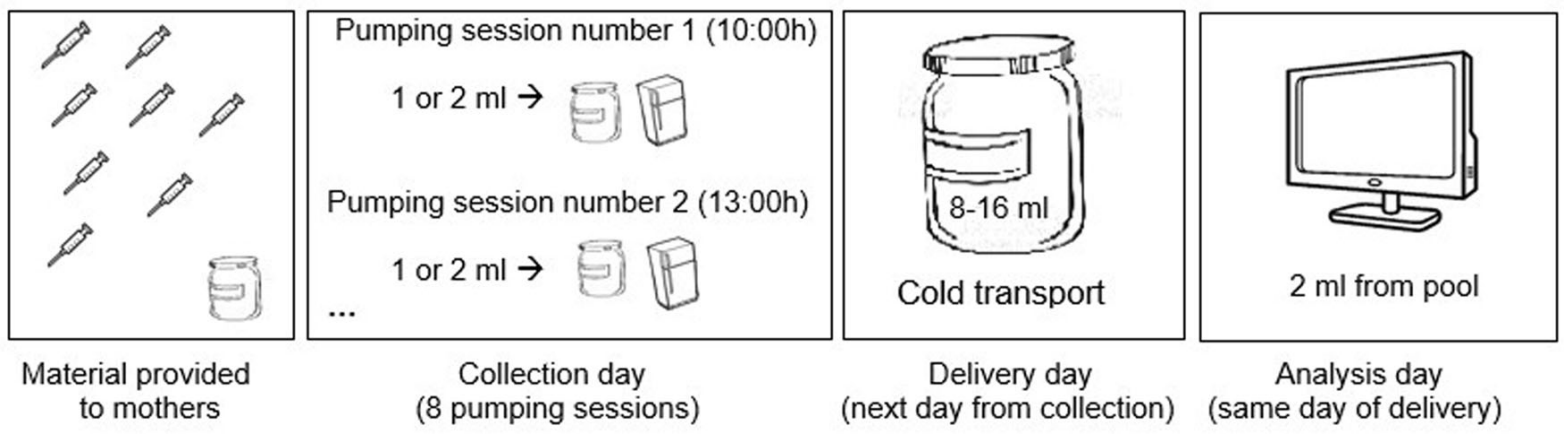
Sample collection process. The image belongs to the instructions given to participating mothers at recruitment.

Representative samples of milk produced in a 24 h period (10am to 10am) were obtained by educating participants upon recruitment for the extraction, collection and conservation of samples. They were instructed to manually homogenized their milk immediately after each pumping session by swirling the bottle, before transferring 1–2 ml with a sterile syringe to an independent container, separated from the milk that was going to be used for feeding. This was repeated after each expression (minimum of 15 min), with a recommendation for 8 or more sessions a day, whether by hand or with the help of a manual or electric pump. Two ml from the pool (8–16 ml in total) were used for milk macronutrient analysis; the remaining milk was frozen at −80 °C for further research related to other aspects of preterm human milk, as showed in Fig. 1. This was repeated on weeks 1, 2, 4 and 8 after delivery or until discharge, whichever was first. On weeks 3, 5, 6, and 7 mothers delivered a single morning sample. Due to the singular biological relevance and the limited volume of colostrum, the first study sample was collected between day 7 and 10 of lactation. Overall composition over the first 4 weeks of life was calculated for each macronutrient as the average of the results of daily pooled samples for weeks 1, 2, and 4.

Macronutrient content was measured with a mid-infrared transmission spectrometer (MIRIS Human Milk Analyzer®, MIRIS solutions, Uppsala, Sweden), following manufacturer´s instructions. MIRIS Human Milk Analyzer® measures lipids and total protein (g/dl) and estimates true protein, carbohydrates (g/dl) and energy (kcal/dl). For this study, true protein (reflects the nitrogen associated with protein) was estimated by subtracting a theoretical 20% non-protein nitrogen from the measured total protein [26] and energy was calculated by the formula: 4.4 kcal* g of total protein + 9.25 kcal* g of fat + 4 kcal* g of carbohydrate [27], and was transformed into kilojoules (kJ). This device has demonstrated repeatability of <0.05% and accuracy of < 0.1% [11] and has been previously shown to be adequate for macronutrient measurement of human milk, although some variability may arise from the presence of human oligosaccharides and non-protein nitrogen and the tecnique is also vulnerable to heterogeneity in sample preparation [28]. Given that some of the limitations arise from sample treatment, efforts were taken to standardize collection and pre-analytical treatment as much as possible and actual measurements were mostly restricted to two trained operators (CBN, MLA), one per site. Milk aliquots were warmed to room temperature, manually homogenized, and subsequently heated up to 40 °C in a thermal block or water bath before injection in the cuvette. We mainly addressed protein and fat/energy content, as the main determinants of preterm growth [22] and used 24 h pooled milk to mitigate the effects of circadian variation. Week 1 and week 4 were assumed to represent early/transitional and mature composition, respectively.

Maternal, obstetric and neonatal demographic and clinical data were obtained from clinical charts and from self-reported information during an interview upon recruitment. Intrauterine growth restriction (IUGR) was defined as estimated foetal weight under percentile 3 or under percentile 10 with placental dysfunction. Hypertensive disorder of pregnancy was defined as maternal blood pressure higher than 140/90 mmHg and increased angiogenic factors or evidence of damage to a target organ (including hepatic, renal, hematological or cerebral disease) not attributable to another cause. Delivery modes were vaginal delivery (which included instrumental delivery) and caesarean section (specifying with or without labour). Labour was defined as the presence of painful uterine contractions together with cervical dilatation.

All data were collected and managed in a database specifically created for this study using REDCap electronic data capture tools hosted at Hospital Sant Joan de Déu, within the REDCap secure platform with codification of participants [29].

Statistical analysis was performed with the SPSS® (Social Package for Social Sciences, IBM Corporation, USA) software, v25. Normality was tested with the Kolmogorov–Smirnov test and by visual inspection of histograms. Pearson and Spearman correlations were used to explore relationships between quantitative variables; differences between groups were analyzed by chi-square tests for categorical variables, Student´s t or ANOVA for continuous variables with normal distribution or non-parametric tests if distribution was not normal. In order to assess the impact of maternal/perinatal conditions on macronutrient content while considering potential confounding effects, we used linear regression models with the macronutrient concentration at a specific time point as the dependent variable and the conditions that reached significance in univariate analysis as independent variables. Differences in macronutrients over time in the whole sample were assessed by a linear mixed model to account for repeated measures and missing data, using an autoregressive heterogeneous covariance structure, and time as a fixed effect, where fixed effects of time (weeks) were categorical. Statistical significance was set at 0.05.

Based on previously reported data on preterm milk composition and accepting an alpha risk of 0.05 and 80% statistical power, we calculated that we would need a minimum sample of 33 patients per group (based on maternal/ perinatal conditions) to detect a difference of 7 kcal/100 mL and of 0.15 g of protein/100 mL, both of which have been previously shown to have an impact on preterm growth [30].

## Results

We recruited 192 mothers delivering very preterm infants at 32^0^ or less week’s gestation during the study period. Of them, 117 women (having delivered 130 VPI) contributed samples with a valid macronutrient reading during hospital stay. All mother-infant/s dyads/tryads were followed up until discharge.

### Maternal and neonatal characteristics

Forty-five mothers (38.8%) were over 35 years old, and more than half (66, 56.4%) were primiparous women. In relation to maternal body mass index, eleven (9.4%) mothers were underweight, twenty-one (17.9%) were overweight and seventeen (14.5%) were obese. Eleven mothers (9.4%) suffered from gestational diabetes.

There were twenty multiple gestations (17.1%). Twenty mothers (17.1%) suffer from hypertensive disorders of pregnancy and 23 mothers (19.7%) were diagnosed with IUGR. About half of deliveries were vaginal (54, 46.2%) and the other half by caesarean section (62–53.0%), of which 25 (40.3%) were elective caesarean sections without labour. Regarding lactation history, 30 women (25.6%) had previously breastfed for a median of 13.4 ± 10.7 months (exclusively for a median of 4.8 ± 2.5 months).

Mean gestational age at delivery was 28.7 ± 2.3 weeks (range 23^3^–32^0^) and mean birth weight 1167 ± 380 grams (range: 460–1900). There were 69/130 (53.1%) boys.

### Global milk macronutrient composition and evolution with lactation time

Infrared technology was used to analyze 648 human milk samples. Twenty-three results (3.5%) were considered outliers and excluded, as they fell outside the measuring range of the device ± reported accuracy [31], leaving 625 valid readings. Three-hundred and nineteen of them (51.4%) correspond to 24 h pools, and Table 1 summarizes their average macronutrient and energy content. Associations between milk composition and time and maternal/neonatal characteristics were explored using these 319 pooled samples in order to avoid the possible effects of circadian rhythms.

**Table 1:** Macronutrient and Energy Concentration in Very Premature Mother´s Milk in the Pooled Samples from Weeks 1, 2, 4, and 8.

	Week 1 (*n* = 94)	Week 2 (*n* = 99)	Week 4 (*n* = 84)	Week 8 (*n* = 42)	*p*-value*	Average concentration in pooled samples from weeks 1, 2, 4, and 8 (*n* = 319)		Average concentration in pooled samples from first 4 weeks (*n* = 277)	
	Mean ± SD	Mean ± SD	Mean ± SD	Mean ± SD		Mean ± SD	Range	Mean ± SD	Range
True protein (g/dl)	1.5 ± 0.3	1.3 ± 0.2	1.1 ± 0.2	1.1 ± 0.2	<0.0001	1.3 ± 0.3	0.7–2.5	1.3 ± 0.2	0.9–2.5
Carbohydrate (g/dl)	7.1 ± 0.6	7.3 ± 0.5	7.5 ± 0.5	7.5 ± 0.5	<0.0001	7.3 ± 0.6	5.6–8.9	7.2 ± 0.5	6.0–8.2
Lipids (g/dl)	3.9 ± 1.1	3.8 ± 1.0	3.4 ± 0.9	3.7 ± 0.9	<0.0001	3.7 ± 1.0	1.1–6.5	3.7 ± 0.9	1.2–6.4
Energy (kJ/dl)	305.6 ± 45.2	300.1 ± 39.3	281.3 ± 35.6	294.3 ± 36.8	<0.0001	296.0 ± 41.0	171.6–414.4	296.8 ± 36.4	170.8–401.9
Energy (kcal/dl)	73.0 ± 10.8	71.7 ± 9.4	67.2 ± 8.5	70.3 ± 8.8	<0.0001	70.7 ± 9.8	41.0–99.0	70.9 ± 8.7	40.8–96.0

SD Standard deviation.

*p-value for the linear mixed effects model for repeated measures per subject with week as fixed effect

**Fig. 2:**
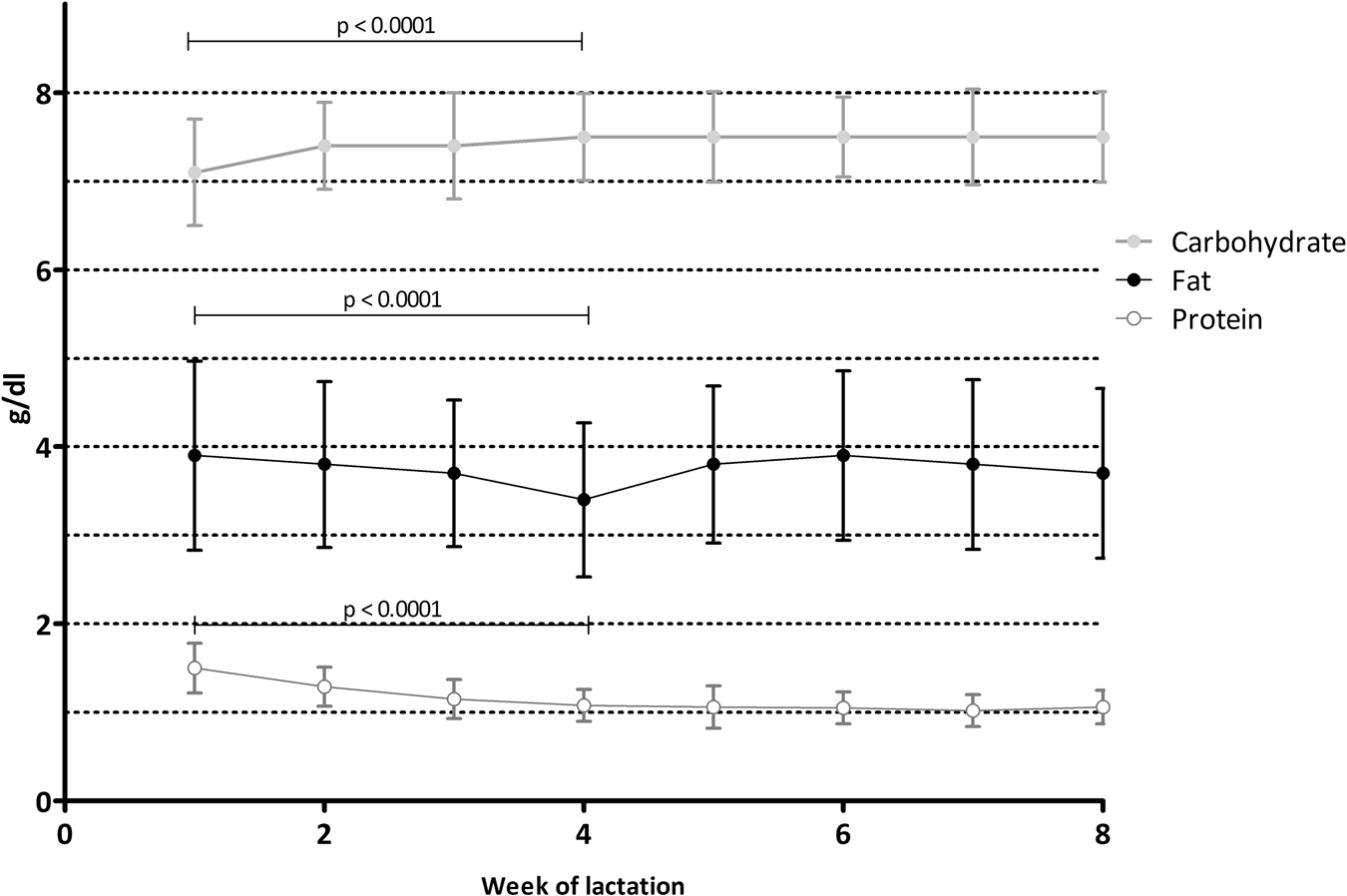
Macronutrient concentration (g/dl) by week of lactation. Circles indicate the mean and error bars represent standard deviation (*n* = 625). Statistical analysis was performed on the results of 24 h pooled samples (weeks 1, 2, and 4, *n* = 277), resulting in significant differences from week 1 to 4(type III test of fixed effects for week: fat: F 9.468, *p* < 0.0001; differences were significant between week 1 and 4: *p* < 0.0001, week 2 and 4: *p* = 0.001, but not between weeks 1 and 2: *p* = 0.199. Protein: F 110.245, *p* < 0.0001, with significant differences between weeks in all paired analysis; Carbohydrate: F 16.918, *p* < 0.0001, with significant differences between weeks in all paired analysis. Energy: F: 11.839, *p* < 0.0001, with significant differences between weeks in all paired analysis).

There was a decrease in fat, protein and energy and an increase in carbohydrate content with length of lactation. Differences were significant from week 1 to 4 when analyzed from the results of 24 h pooled samples (weeks 1, 2, and 4), with significant differences between the 3 time points in all-paired analysis for protein, carbohydrate and energy and between weeks 1 and 4 for fat (Fig. 2). We did not attempt to analyze later weeks due to a much smaller sample size per time point.

### Macronutrient concentration in milk in relation to maternal, gestational and neonatal characteristics

Table 2 summarizes statistically significant results of univariate analysis of associations between maternal/perinatal conditions and preterm milk macronutrient content in the 24-hour pooled samples at different time points during lactation.

**Table 2:** Results of univariate analysis of associations between maternal and gestational conditions and preterm milk macronutrient content at different time points (*n* = 277).

Nutrient	Condition		Time point		Concentration	95% CI of difference	*p*-value
Protein in g/dl	Maternal	Age (years old)	Week 1	<35	1.5 ± 0.3	0.005–0.240	0.043
				≥35	1.6 ± 0.3		
			1^st^ 4 weeks of lactation	<35	1.3 ± 0.2	0.023–0.211	0.022
				≥35	1.4 ± 0.3		
		BMI (kg/m^2^)	Week 4	<25	1.1 ± 0.2	0.020–0.183	0.010
				≥25	1.2 ± 0.2		
	Gestational	Multiple	Week 1	Yes	1.4 ± 0.3	0.007–0.302	0.003
				No	1.5 ± 0.3		
			Week 4	Yes	1.0 ± 0.1	0.098–0.221	<0.001
				No	1.1 ± 0.2		
			1^st^ 4 weeks of lactation	Yes	1.2 ± 0.3	0.004–0.244	0.001
				No	1.4 ± 0.2		
		IUGR	Week 4	Yes	1.2 ± 0.2	(−0.230)-(−0.045)	0.010
				No	1.1 ± 0.2		
			1^st^ 4 weeks of lactation	Yes	1.4 ± 0.3	(−0.235)-(−0.006)	0.047
				No	1.3 ± 0.2		
		Hypertensive disorders	Week 4	Yes	1.2 ± 0.2	(−0.221)-(−0.015)	0.043
				No	1.1 ± 0.2		
	Labour and delivery	Labour	Week 4	Yes	1.1 ± 0.2	0.051–0.229	0.004
				No	1.2 ± 0.2		
	Lactation history	Previous lactation	1^st^ 4 weeks of lactation	Yes	1.3 ± 0.3	(−0.028)−0.192	0.029
				No	1.4 ± 0.2		
Fat in g/dl	Maternal	BMI (kg/m^2^)	Week 4	<25	3.2 ± 0.8	0.067–0.907	0.018
				≥25	3.7 ± 0.9		
	Gestational	Multiple	Week 4	Yes	3.0 ± 0.0	0.22–0.925	0.018
				No	3.5 ± 0.9		
		Hypertensive disorder of pregnancy	Week 1	Yes	3.3 ± 1.2	0.116–1.267	0.019
				No	4.0 ± 1.0		
Energy in kJ/dl (in kcal/dl)	Maternal	BMI (kg/m^2^)	Week 4	<25	274.2 ± 34.7 (65.6 ± 8.3)	0.663–8.676	0.023
				≥25	293.9 ± 35.6 (70.2 ± 8.5)		
	Gestational	Multiple	Week 4	Yes	262.9 ± 22.2 (62.8 ± 5.3)	2.412–9.266	0.001
				No	285.9 ± 36.4 (68.3 ± 8.7)		
			1^st^ 4 weeks of lactation	Yes	286.3 ± 36.4 (68.4 ± 8.7)	(−1.244) −7.255	0.043
				No	298.9 ± 36.4 (71.4 ± 8.7)		
		Hypertensive disorder of pregnancy	Week 1	Yes	279.6 ± 52.7 (66.8 ± 12.6)	0.243–14.286	0.014
				No	309.8 ± 42.3 (74.0 ± 10.1)		

#### Maternal conditions

Advanced maternal age was related to higher milk protein concentration (Table 2), without differences in energy content. There was a weak positive correlation between maternal age and week 1 and week 2 milk protein concentration (*r* = 0.216, *p* = 0.037; *r* = 0.322, *p* = 0.001, respectively).

Overweight and obese mothers had higher protein, fat, and energy content in mature (week 4) milk (Table 2), with no differences in overall composition over the first 4 weeks.

#### Pregnancy characteristics

Nutritional content was lower in the milk of mothers delivering multiples, both in early lactation, mature milk and average protein and energy content over the first 4 weeks (Table 2).

Mothers with hypertensive disorders of pregnancy produced early milk with lower fat and energy content, but the contrary was true regarding week 4 protein, which was higher in mothers with pre-eclampsia/eclampsia (Table 2).

Protein concentration was higher in milk from mothers of singleton IUGR babies, both at week 4 and over the first 4 weeks of lactation (Table 2).

We found no differences between mothers that had and had not experienced gestational diabetes.

#### Labour and delivery

There were no differences in milk composition between mothers who underwent caesarean section and those who did not, but women who had been in labour had lower protein in week 4. There were no detectable differences in the average protein and energy content over the first 4 weeks.

#### Lactation history

Average protein content over the first 4 weeks of lactation was lower in women who had previously breastfed. There was a positive moderate correlation between duration of previous lactation (in months) and protein content during the first 4 weeks of lactation (*rho*: 0.436, *p* = 0.029).

#### Neonatal characteristics

Gestational age was negatively weak correlated with protein content on week 1 (*rho*:−0.337, *p* < 0.001), week 4 (*rho*:−0.217, *p* = 0.036) and average protein content during the first 4 weeks (*rho*:−0.307, *p* < 0.001). It also negatively weak correlated with fat (r:−0.202, *p* = 0.038) and energy (r:−0.229, *p* = 0.018) on week 1 and with average energy content during the first 4 weeks (r: −0.193, *p* = 0.003). There were no differences according to neonatal gender.

### Multivariate analysis

The only independent predictors of early milk protein and energy (week 1) content were gestational age and having suffered a hypertensive disorder of pregnancy. Protein concentration at 4 weeks was independently influenced by multiple gestation, maternal pre-pregnancy BMI higher than 25 and the presence of labour before birth. Energy content on week 4 was related to both maternal overweight/obesity and hypertensive disorders of pregnancy.

**Fig. 3:**
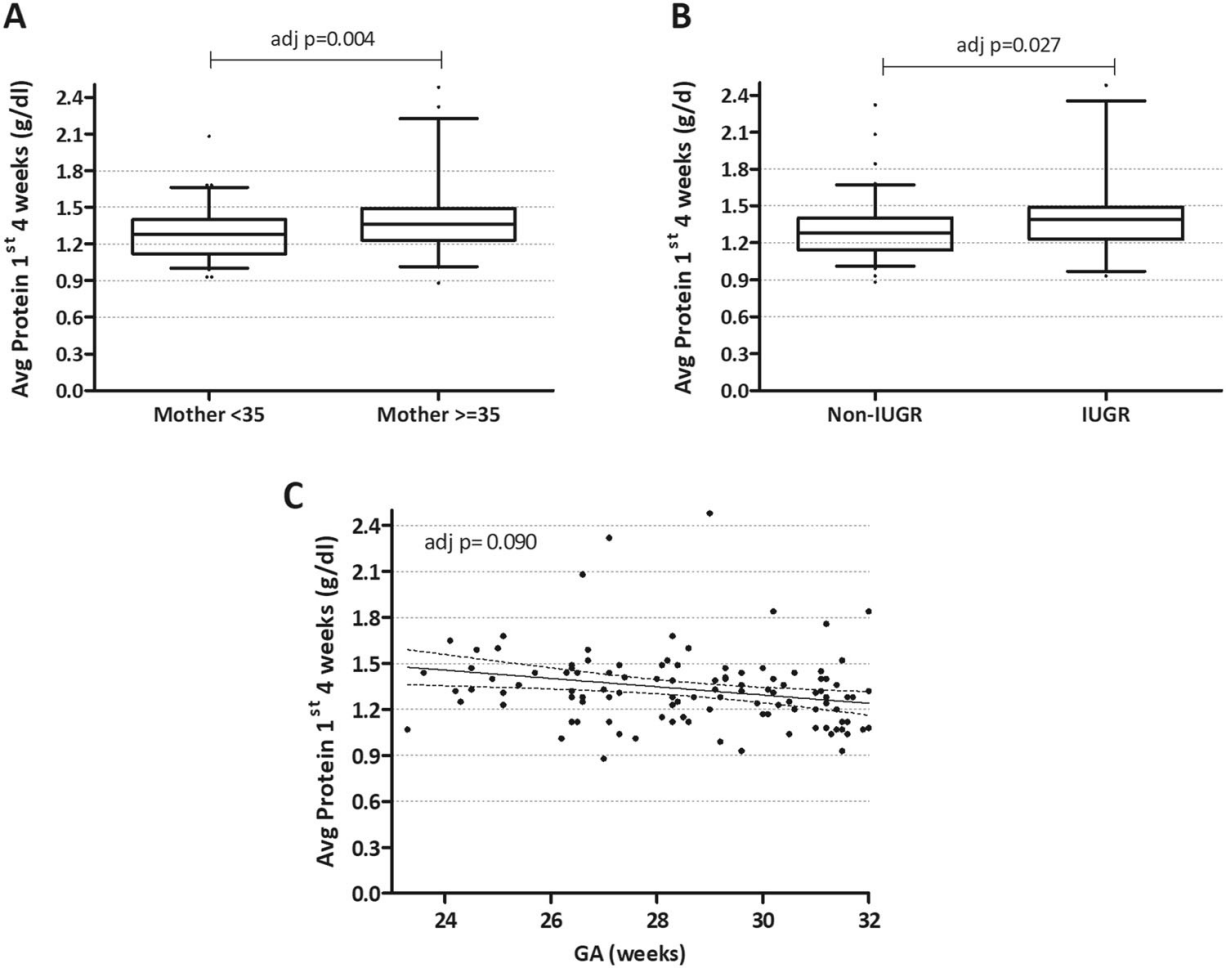
Factors associated with average protein concentration over the first 4 weeks of lactation (g/dl) in multivariate analysis. The average protein concentration was calculated based on pooled samples from weeks 1, 2 and 4 (*n* = 277). **A**, **B** Boxplots comparing mean concentration between groups by maternal age and presence of IUGR, with boxes outlined between the first and the third quartile, and a horizontal line in the median. The whiskers span between the 5^th^ and the 95^th^ centile. **C** Scatterplot showing average protein content over the first 4 weeks against gestational age at birth. Best-fit regression line with 95% CI is also shown.

The best model for predicting average milk protein content over the first 4 weeks included older maternal age (35 years or older), duration of pregnancy and presence of IUGR (Fig. 3). We could not find a combination of maternal and pregnancy characteristics which were predictive of milk energy content over the first 4 weeks. Final regression models are summarized in Table 3.

**Table 3:** Multivariate analysis of perinatal factors influencing maternal milk composition by linear regression. Best fitting models were selected by the backwards method, with a p-value cut-off of 0.100, after inputting all variables with statistical significance in the univariate analysis.

Time point	Nutrient	Best Model		Coefficients	Standardized β coefficients	Adjusted *p*-value
		Adjusted R^2^	*p*-value			
Week 1	Protein^a^	0.051	0.018	Gestational age	−0.247	0.018
	Energy^b^	0.055	0.014	Hypertensive disorder of pregnancy	−0.257	0.014
Week 2	Protein^c^	0.085	0.002	Maternal age (years)	0.307	0.002
Week 4	Protein^d^	0.326	<0.0001	Multiple gestation	−0.227	0.024
				Maternal BMI ≥ 25	0.286	0.005
				Labour before birth	−0.447	<0.0001
	Energy^e^	0.083	0.016	Maternal BMI ≥ 25	0.257	0.023
				Hypertensive disorder of pregnancy	0.209	0.062
1st 4 weeks of lactation	Protein^f^	0.113	0.002	Maternal age ≥35 years	0.272	0.004
				IUGR	0.209	0.027
				Gestational age	−0.160	0.090

Initial factors in the models: ^a^Protein week 1: multiple pregnancy, Hypertensive disorder of pregnancy, maternal age, gestational age / ^b^Energy week 1: Hypertensive disorder of pregnancy, presence of labour, gestational age, alcohol consumption / ^c^Protein week 2: multiple pregnancy, maternal age (years), gestational age / ^d^Protein week 4: multiple pregnancy, IUGR, Hypertensive disorder of pregnancy, presence of labour, gestational age, maternal BMI < 25 or ≥ 25 kg/m^2^ / ^e^Energy week 4: multiple pregnancy, Hypertensive disorder of pregnancy, maternal BMI < 25 or ≥25 kg/m^2^, smoking / ^f^Average protein over the 1st 4 weeks of lactation: multiple pregnancy, Hypertensive disorder of pregnancy, maternal age < 35 or ≥35 years old, gestational age, IUGR, previous breastfeeding.

## Discussion

Our results on global macronutrient (protein, carbohydrate, fat) concentrations are in line with those from similar populations [12, 16, 22, 32]. Fat content was the most variable, with up to 6-fold concentration difference between samples, followed by protein (4-fold concentration range), as previously reported [32, 33].

### Evolution of milk composition with lactation time

We have confirmed previous findings suggesting that macronutrient composition of preterm human milk changes over time, with an inverse relationship between protein content and days of lactation. In contrast with other reports [13, 16, 18], average protein concentration in our population remains above 1 g/dl at least for the first 8 weeks. Even so, milk from mothers included in our study would fail to meet protein intake recommendations [8] after standard fortification at 160 ml/kg/day of volume intake in 3 out of 4 samples for patients under 1000 g. Arslanoglu et al. [34] observed a consistent deficit between 0.5 and 0.8 g/dl when comparing estimated and measured protein intake of fortified human milk [34]. Regarding fat, most studies describe an increase in content over time [10, 17, 18, 35], but they generally include a first measurement within the first days of lactation, while our schedule would miss the lipid increase from colostrum to transitional milk (first sample on day 7). In our samples, fat decreased over the first month and then increased. Although some authors report erratic distributions of fat content [13, 18], we think that, in our case, it is likely that women contributing samples from week 4 onwards were mothers to infants of lower gestational ages, who, as seen, tend to have milk with a higher fat content.

### Macronutrient concentration in milk in relation to maternal characteristics

#### Maternal age

Some differences have been reported regarding fat content in relation to maternal age [11, 36–38], but not protein concentration. This might be due to heterogeneous target populations, sampling or cut points for the definition of advanced maternal age. Older mothers might also carry a higher risk of obstetric complications like gestational diabetes and preeclampsia, which might act as confounders [39, 40], although this was not the case in our sample.

#### Maternal BMI

Most previous studies find differences similar to ours in milk macronutrient at different points in the first month of lactation between obese and non-obese mothers, particularly regarding a higher fat content, and occasionally also a higher protein content [11, 13, 14, 17, 37, 41]. It has been speculated that this could be the result of higher substrate availability due to increased serum concentrations of free fatty acids and amino acids in obese women [14] or relate to other metabolic abnormalities [11, 42].

### Macronutrient concentration in milk in relation to obstetric characteristics

#### Multiple pregnancy

Multiple birth has seldom been considered when studying milk composition. Congiu et al. described a higher protein and lower fat content in a small sample of preterm multiples, while our results point to a lower concentration of protein, fat and energy in mature milk of mothers of multiples [43]. The much higher proportion of small for gestational age in the multiples in the Italian study (18.6 vs 3.7%) might partially explain the discrepancy. Lower macronutrient concentration has been related to higher milk yields [44]. We unfortunately do not have data on milk production from our participants, but there might be a selection bias due to the criteria of milk volumes been enough to cover for infant feeding requirements, which will obviously be higher when providing for more than one baby.

#### Pregnancy complications

In agreement with our results, a lower earlier fat content and a higher protein density after the first few weeks following preterm birth in mothers with a history of preeclampsia [45] have been reported. The relationship between suboptimal fetal growth and human milk macronutrients is controversial, likely due to heterogeneity in definition of IUGR and in the gestational age of target populations. The aforementioned study by Correia et al. described a weak negative association of IUGR with fat but not protein content during the first weeks, which is in disagreement to our findings [45]. This may be due to differences in other characteristics (maternal BMI, breastfeeding history) that we have found to have an association and were not included in their analysis.

#### Labour and delivery

It is biologically plausible that physiologic changes associated with labour, rather that the final delivery route, might change milk composition in the same way it has been shown to impact the maternal hormonal response to breastfeeding (especially oxytocin and prolactin levels) [46]. In our data, the presence of labour related to a lower concentration of protein in week 4, in line with other authors [13, 36]. Results are more controversial regarding early milk [13, 38], and other studies [32] find no differences at all at any time point.

#### Lactation history

In agreement with another cohort of mothers delivering very prematurely [37], women who had previously breastfed had lower protein concentration during the first month. It has been hypothesized that this is due to a faster onset of type II lactogenesis and an increased milk production [37]. Surprisingly, within this subgroup of mothers we also see a positive correlation between duration of previous lactation and milk protein content during the first 4 weeks after delivery. Although it may seem unexpected, it has been reported that protein content increases in prolonged (more than 12 months) or in tandem lactations [47]. It could be speculated that duration of previous lactation or a shorter interlactation period (as would be expected to be associated with longer periods of breastfeeding an older sibling) might have an impact on subsequent milk composition, although we could not find any previous research on this topic.

### Macronutrient concentration in milk in relation to neonatal characteristics

We found an inverse relationship between gestational age and protein concentration during the first month of lactation, as reported by most authors [11, 37, 43, 48]. This has been proposed to be the result of mammary gland immaturity with a reduced in blood flow, incomplete differentiation of epithelial cells and an absence of junctions [49, 50].

Our study has some limitations. Macronutrient analysis of the samples were performed uniquely. Some studies confirm their results by performing duplicate analysis [32] or by checking the results with classical methods [26]. Even efforts were made to ensure identical methodology; results may be influenced by center. Our results do not differentiate between colostrum and transitional milk.

According to the findings of our observational study, gestational age negatively correlated with both protein and energy content of milk, and younger mothers, with lower BMI, having non-IUGR babies and multiples might have lower average protein content during the first weeks of lactation. Neonatologists should be aware that intake calculations based on general estimations of milk macronutrients may well be inaccurate and standard fortification may leave some patients well under the high protein intake requirements of VPI [51]. Identifying mothers at risk of having lower protein or caloric milk density could help guide more personalized nutritional interventions [16, 51, 52], like adjusted, targeted or double fortification, as cumulative protein and caloric intake deficits over the first few weeks of life of a VPI will negatively affect postnatal growth [2]. Although changes in composition were numerically small, some were consistent over the first month of lactation, so that any impact on neonatal growth would be amplified when calculating cumulative deficits [2]. Our study supports the need for nutritional analysis of human milk that could be focused on children with poor postnatal growth or on mothers with factors associated to production of milk with lower nutritional density.

## Data Availability

The datasets generated and analyzed during the current study are available from the corresponding author on reasonable request.
